# Investigation on the Effect of Oral Breathing on Cognitive Activity Using Functional Brain Imaging

**DOI:** 10.3390/healthcare9060645

**Published:** 2021-05-29

**Authors:** Ju-Yeon Jung, Chang-Ki Kang

**Affiliations:** 1 Department of Health Science, Gachon University Graduate School, Incheon 21936, Korea; 95me@naver.com; 2 Neuroscience Research Institute, Gachon University, Incheon 21565, Korea; 3 Department of Radiological Science, College of Health Science, Gachon University, Incheon 21936, Korea

**Keywords:** oral breathing, brain health care, working memory, 2-back working memory, functional MRI

## Abstract

Oral breathing directly affects behavioral performance and dental health. Various relationships between oral breathing and periodontal disease have been well-described. However, the effect of oral breathing on cognitive performance remains unclear. This study aimed to investigate the effects of oral breathing on cognitive function using functional magnetic resonance imaging (fMRI). Twenty-two healthy participants (mean age, 22.27 ± 1.42 years) performed a two-back (2B) working memory fMRI task using a 3T MRI scanner while breathing through their oral or nasal passage. Functional activity analysis was performed using a statistical parametric mapping software package. One-sample group analyses were performed in 2B > Rest contrast. Functional connectivity analysis was conducted using MATLAB-based imaging software. Mixed ANOVA analysis was performed. The results showed more brain activation and connection during nasal breathing than during oral breathing. For Nasal > Oral contrast, various functional connections are known to have a significant relationship with working memory, including the left cerebellum, left and right inferior parietal gyrus. This can be significant evidence to demonstrate that oral breathing is an inappropriate method for intellectual activity using brain imaging techniques. Therefore, this study suggests that changing various habits related to oral breathing is important for cognitive function.

## 1. Introduction

Oral breathing can be induced in various situations, such as nasal congestion due to hypertrophic adenoids and/or allergic rhinitis, even in healthy individuals. In general, oral breathing reaches approximately 17% even in healthy subjects, as patterns of nasal congestion and relief appear in daily life [1]. Real problems with oral breathing can affect health in several ways. Fundamentally, oral breathing is a risk factor for dental health. During oral breathing, malocclusion may occur, the intraoral space becomes dry and saliva production is reduced. Saliva has several important roles in dental health, such as protection against the risk of tooth decay and periodontal disease [2,3]. Furthermore, prolonged oral breathing can cause not only dental disease but it can also cause physical changes such as imbalance of craniofacial muscle activity and deformation of head posture produced by positional changes of the tongue, lips, and mandible [4,5,6,7]. Oral breathing can also affect pulmonary function. Lung function and respiratory muscle activity can be altered by other mechanisms of oral breathing [8]. Failure in filtering, humidifying and warming of the inhaled air through oral breathing may lead to decreased lung function [9]. For these reasons, interest, care and research on oral breathing are important for human health.

Furthermore, previous studies have observed that oral breathing can increase the likelihood of brain functional problems due to lower oxygen saturation in the human brain [10,11]. Recent studies have demonstrated an association between oral breathing and cognitive deficits [12,13,14]. Significant decreases in memory and learning ability during oral breathing, and changes in the central nervous system, have been reported in animal research [14]. In addition, there was a decline in working memory performance for specific cognitive tasks in children with oral breathing, and for olfactory memory task caused by oral breathing in healthy adults [13,15]. Furthermore, changes in brain function, including oxygen load and brain activity during oral breathing, have been demonstrated in a variety of ways [12,16,17,18]. Recently, brain wave spectrum analysis using electroencephalography (EEG) and blood oxygenation level-dependent (BOLD) functional magnetic resonance imaging (fMRI) techniques were introduced to investigate the breathing effect [12,18,19]. Previous studies have shown that oral breathing could change the default mode network and create more widespread brain functional connectivity in oral breathing conditions than in nasal breathing conditions during the resting state, and brain activities during oral inhalation and exhalation substantially differed from those during other respiration patterns, especially in the hippocampus and brainstem [18,19]. On the other hand, oral breathing can change theta and alpha power activity during working memory tasks by decreasing oxygen saturation [12]. However, there is little evidence regarding the influence of oral breathing on functional brain activity during cognitive tasks.

Therefore, it is important to clearly understand the role of breathing in the human brain to live a healthy life. Although various effects of oral breathing have influenced human life, there is little evidence to investigate brain activity induced by working memory tasks. It is necessary to understand cognitive changes in oral breathing via working memory tasks because they can be a major cognitive element highly related to academic ability and can play an important role in attention, short-term memory and concentration [20]. Therefore, the main objective of this study was to determine the effect of oral breathing on brain function. Therefore, the hypothesis for this study was that oral breathing affects working memory performance or brain functional activity. The detailed objectives were to compare the differences in active brain regions and functional connectivity between nasal (normal) and oral breathing during a two-back (2B) working memory task using BOLD fMRI.

## 2. Materials and Methods

### 2.1. Participants and Data Acquisition

Twenty-two healthy volunteers (12 women and 10 men; mean age, 22.27 ± 1.42 years) participated in the study after signing an informed consent form. The study protocol was approved by the institutional review board (IRB number: GDIRB2017-174). The procedure was conducted in accordance with the approved guidelines. The participants had no history of neurological, psychiatric, or respiratory disorders.

In the fMRI experiments, all participants underwent three imaging sessions on the same day: one brain structure scan and two fMRI scans for the working memory task during oral and nasal breathing. The experiment was performed using a 3T MRI scanner (Siemens Verio, Erlangen, Germany) with a commercially available 12-channel radiofrequency head matrix coil for whole-brain imaging. The sequences included: 1) high-spatial-resolution T1-weighted anatomical three-dimensional (3D) imaging with magnetization–prepared rapid acquisition gradient echo (MP-RAGE) with repetition time (TR) of 1900 ms, inversion time (TI) of 900 ms, echo time (TE) of 2.93 ms, flip angle (FA) of 9°, 176 slices, field of view (FOV) of 256 mm, 1 × 1 × 1 mm^3^ (interpolated to 0.5 × 0.5 × 1 mm^3^) isotropic resolution, and acquisition time (TA) of 3 min 29 s; and 2) BOLD fMRI sequence of two-dimensional (2D) echo planar imaging (EPI) with TR: 2000 ms, TE: 30 ms, in-plane resolution: 3.05 × 3.05 mm^2^, slice thickness: 3 mm (with a gap of 1.5 mm), FOV: 195 mm, 30 slices, 199 volumes, and TA: 6 min 38 s.

### 2.2. Breathing Control

Every participant practiced becoming familiar with oral breathing until the participants could naturally switch their breathing pattern from nasal breathing. During the oral breathing session, participants used a nasal plug to induce oral breathing naturally without any enforcement. A plug was necessary for all subjects to avoid any additional sensations. During the nasal breathing session, participants were asked to close their mouths to eliminate any interference from oral breathing. To stabilize the respiratory effect, training was performed to check whether the abdominal volume change and respiratory rate were kept constant during breathing exercises before MRI scanning [21,22]. We also trained the participants to maintain a normal breathing range before the experiment. During the experiment, they were asked to maintain a normal breathing rate (12–20 repetitions/min) rather than deep breathing.

### 2.3. Stimulation Paradigm for fMRI

The participants were asked to perform a 2B working memory task. An experimental design was used in which three alternating working memory tasks (three blocks) and three rest periods were mixed. Each working memory task block consisted of 15 stimuli (trials), and a total of 45 stimuli for three blocks were presented in each breathing session. The stimulus protocol of 2 s consisted of a white number from 0 to 9, with a presentation duration of 500 ms, followed by a white fixation cross for 1500 ms, so that each working memory block lasted for 30 s (see Supplementary Figure S1). The order of breathing (oral or nasal) was controlled randomly to eliminate order effects.

The block and stimuli order for each condition was maintained constant for all participants. During the sessions, participants were required to pay attention to pressing a button with their right index finger within a short period following a visual cue presented on a mirror through a projector beam. The task paradigm was built using DMDX software (http://www.u.arizona.edu/~kforster/dmdx/dmdx.htm, accessed on 7 July 2017). The response time and accuracy of each trial were recorded [23]. Accuracy was calculated using the following formula: number of trials with correct answers/total number of trials × 100.

### 2.4. Data Analysis

For brain functional activity analysis, all data were analyzed using SPM12 (http://www.fil.ion.ucl.ac.uk/spm, accessed on 2 February 2019). We acquired a total of 199 volumes of image data, and each volume consisted of 30 slices covering the entire brain. Before preprocessing the fMRI data, we discarded the first four volumes (8 s) out of 199 volumes on each breathing protocol to ensure only the collection of stabilized data. In the preprocessing analyses, 195 functional volumes were utilized for functional analysis. Therefore, they were realigned to the first volume to remove rigid-body motion (see Supplementary Tables S1 and S2); slice timing correction, coregistration, and segmentation of high-resolution T1-weighted images were then performed. All images were normalized and spatially smoothed with an 8-mm full width at half-maximum (FWHM) Gaussian kernel to reduce noise. Voxel-wise first-level statistical parametric maps were then generated for individuals using 2B > Rest contrasts. The resulting contrast images during each oral and nasal breathing session for all participants were analyzed using a one-sample *t*-test for second-level random-effect group analysis. The *t*-map results were overlaid on a single-subject T1 template using the SPM tool package. A one-sample *t*-test was performed for each contrast with a threshold of *p* < 0.05, family wise error (FWE) with an extent threshold of 20 voxels. The activated cortical areas for every contrast were expressed using the local maxima labels for automated anatomical labeling (AAL3) [24].

Furthermore, task-related seed to voxel functional connectivity analysis was performed using the CONN toolbox, which is MATLAB-based software (www.nitrc.org/projects/conn, accessed on 21 May 2021). In the preprocessing analysis, functional realignment to the first volume, coregistration, segmentation, normalization and smoothing were performed by a SPM preprocessing pipeline. Denoising was performed with a linear regression and band pass filter (0.008–0.09 Hz) to remove confounding effects. The result of seed-based functional connectivity for each subject were generated by Pearson’s correlation coefficient (first level analysis). For two (breathing) × two (rest and task) mixed ANOVA analysis, gPPI (generalized psychophyisiological interaction) analysis was performed using the first level analysis. Seeds were selected through the results of one-sample *t*-test. In this study, we used 15 seeds in Nasal > Oral contrast (right inferior parietal gyrus, right caudate nucleus, right insula, right cerebellum, right precentral gyrus, right middle frontal gyrus, Vermis, left insula, left precentral gyrus, left inferior parietal gyrus, left inferior occipital gyrus, left supplementary motor area, left cerebellum, left middle frontal gyrus, left putamen), and 10 seeds were used in Oral > Nasal contrast (right inferior parietal gyrus, right putamen, right cerebellum, right superior frontal gyrus, left inferior parietal gyrus, left insula, left middle frontal gyrus, left supplementary motor area, left precentral gyrus, left inferior frontal gyrus triangular part). A 2 × 2 mixed ANOVA test for each contrast was performed with a cluster threshold FDR-corrected *p* < 0.05 and voxel threshold uncorrected *p* < 0.001. The nonparametric Wilcoxon signed-rank test was used to compare the response time and accuracy during both breathing conditions for all participants, with a significance level of *p* < 0.05, using the statistical tool package (SPSS, version 23; IBM, Armonk, NY, USA).

## 3. Results

The accuracy and response times for each breathing condition were measured using DMDX software. Median accuracy and response time did not show any significant differences between nasal and oral breathing conditions in the 2B task (Table 1).

**Figure 1:**
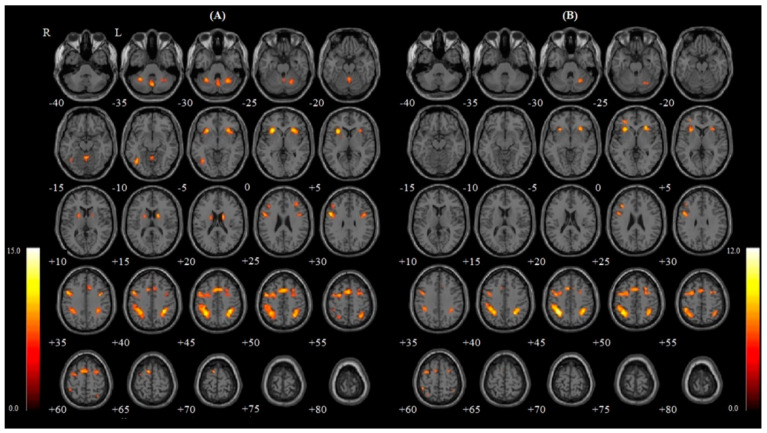
Each breathing condition: (**A**) Nasal and (**B**) Oral for 2B > Rest contrast. Significant activity is shown in brain areas in the axial section view (−40~80, increment 5 mm). The color bar indicates *t* values. R, right; L, left; 2B, 2-back.

Table 2 and Figure 1 show the activation areas in the 2B > Rest contrast for each breathing condition. In the 2B > Rest contrast, 15 regions were activated during nasal breathing and 10 regions were activated during oral breathing. Among these, there were seven common activated regions in both breathing types (Table 2). The right inferior parietal gyrus in the nasal breathing condition had the strongest activation in both breathing conditions (peak z-score in nasal breathing condition: 7.15, *p* < 0.05). Among 15 regions during nasal breathing, five regions (inferior parietal gyrus, insula, cerebellum, precentral gyrus and middle frontal gyrus) appeared in both hemispheres. During oral breathing, however, only one common region (inferior parietal gyrus) appeared (Table 2).

**Figure 2:**
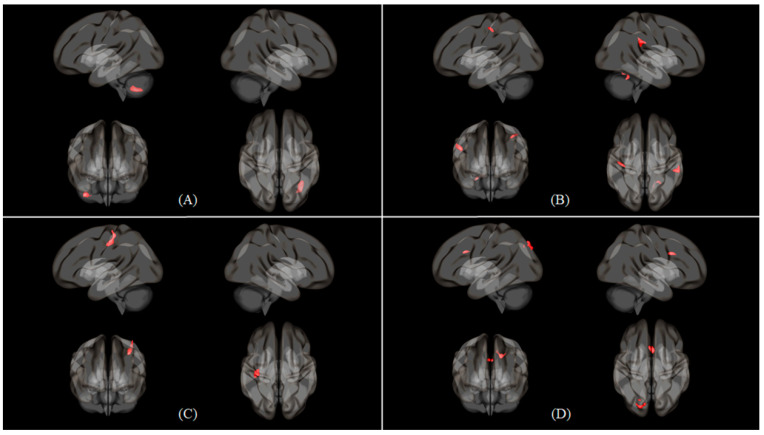
Seed to voxel 3D rendering maps of four seeds in Nasal > Oral contrast with 2B > Rest condition, which were presented in the left, right, anterior and superior views. (**A**) Seed to voxel functional connectivity result of cerebellum 6 (L) seed. (**B**) Inferior parietal gyrus (R) seed. (**C**) Inferior parietal gyrus (L) seed. (**D**) Middle frontal gyrus (L) seed. The maps were obtained at a cluster size threshold (FDR) of *p* < 0.05 with one-sided positive contrast.

**Figure 3:**
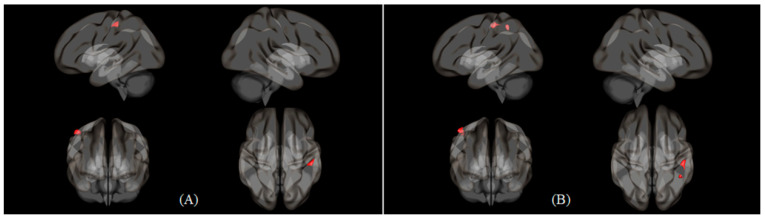
Seed to voxel 3D rendering maps of two seeds in Oral > Nasal contrast with 2B > Rest condition, which were presented in the left, right, anterior and superior view. (**A**) Seed to voxel functional connectivity result of inferior frontal gyrus, triangular part (L) seed. (**B**) Middle frontal gyrus (L) seed. The maps were obtained at a cluster size threshold (FDR) of *p* < 0.05 with one-sided positive contrast.

Table 3 shows the functional connectivity of Nasal > Oral and Oral > Nasal conditions in 2B > Rest. In Nasal > Oral condition, four seeds among 15 seeds had significant functional connectivity. Left cerebellum 6 seed has the strongest connectivity in both condition (cluster size: 240, *p* < 0.05; peak z-score: 4.771, *p* < 0.001). Right inferior parietal gyrus seed had the most connection with other regions (right parietal operculum cortex, left postcentral gyrus, right cerebellum 6) in both conditions (Figure 2B). In Oral > Nasal condition, two seeds among 10 seeds had significant functional connectivity. Both 2 seeds (left inferior frontal gyrus triangular part, left middle frontal gyrus) had common functional connection with postcentral gyrus. The left inferior frontal gyrus triangular part had more functional connectivity peak z-score (4.131) than other seeds. The left middle frontal gyrus had more voxels, which covers the right postcentral gyrus, than other seeds (cluster size of 215) (Figure 3). Furthermore, the left middle frontal gyrus was the only seed that was commonly activated in both conditions during 2B > Rest.

## 4. Discussion

In this study, we attempted to determine the effect of oral breathing on working memory. Thus, we conducted a 2B working memory task during nasal and oral breathing conditions in healthy participants, and their neuronal activity changes were measured using fMRI. As a result, the activity in 15 regions was significantly increased during nasal breathing and 10 regions was increased during oral breathing. Furthermore, the seven functional connections between seeds and voxels were significantly activated in nasal breathing, although only two functional connections were activated in oral breathing.

Table 2 show the activated brain regions during the 2B working memory task according to breathing, presenting a distinct difference in the brain activity pattern between nasal and oral breathing. During oral breathing, activity in some regions (caudate nucleus, inferior occipital gyrus) related to normal working memory disappeared, but unexpected activity appeared in the superior and inferior frontal gyri independent of working memory. In particular, the caudate, which is associated with working memory tasks, has only been discovered in nasal breathing conditions [25]. The absence of activation in the caudate may be related to any change in breathing pattern through the oral cavity because the region is greatly associated with respiratory sensation [26]. Furthermore, in the nasal breathing condition, 10 regions among 15 symmetrically appeared in both hemispheres. However, in the oral breathing condition, only the right and left inferior parietal gyrus were symmetrically activated in both hemispheres (Table 2). Previous studies found trends similar to the current results, in which symmetrical regions were positively activated during nasal breathing [19,27,28]. As in previous research, it is possible that differences in oral and nasal breathing sensation contributed to changes in functional activation.

There were seven common activated regions which were known to be significantly related to working memory task in both breathing condition (Table 2). Among them, four regions had more significant functional connectivity than oral breathing, but one region was included in Oral > Nasal condition. The left cerebellum and the right cerebellum connectivity in Nasal > Oral condition had the most significant connection (Figure 2A). The cerebellum is known to be involved in simultaneous visual- and motor-induced tasks during working memory performance and executive information processing [25,28,29,30,31]. Moreover, the cerebellum was one of significant seeds related to oral breathing. When comparing nasal and oral breathing functional connectivity, cerebellum connections appeared only in nasal breathing conditions [19]. The present results show that cerebellar functional connection is more active in nasal breathing than in oral breathing, similar to recent reports of respiratory changes affecting cerebellar responses [32,33]. In addition, the right inferior parietal gyrus had functional connections with three regions in the Nasal > Oral condition. This is possibly due to the activation of inferior parietal gyrus by nasal breathing and the working memory task. According to the previous research, the inferior parietal gyrus was activated more during nasal breathing than oral breathing [33], and the inferior parietal gyrus had phonological storage role during the working memory task [34]. Thus, we can conclude that various brain regions related to working memory and normal breathing are adequately induced in nasal breathing conditions, but not in oral breathing conditions. This result is similar to that of a previous study in which oral breathing decreased the power of brain waves related to working memory function [12].

Oral breathing infringes on oral health and quality of life, and even interferes with normal brain activity; however, so far, little research has been done in this area. This study provides evidence for the impact of oral breathing and the importance of conventional nasal breathing in working memory function. This demonstrates that breathing through the oral cavity may adversely affect functional brain activity. In a previous study, oral breathing induced more widespread functional connectivity in the resting state [19] and reduced functional activity in the cognitive state. It could be inferred that when people kept breathing orally, the brain had improper activation during resting and intellectual state. Therefore, controlling various factors that induce nasal obstruction (humidity, body weight, allergy, etc.) to maintain ideal nasal space is important for intellectual function, especially for children and adolescents during brain growth.

However, this study has several limitations. Longitudinal studies are necessary in patients with prolonged oral breathing habits to verify its long-term effects. The short-term breathing method used in this study was not like the regular breathing habits of healthy adults. Because most participants are accustomed to breathing through the nasal cavity, it is relatively difficult for them to perform a cognitive task during oral breathing. When comparing oral breathing patients and nasal breathing healthy subjects, the difference in brain activity caused by breathing may be even greater. Furthermore, since oral breathing patients have problems with dental health, it is also important to observe changes in brain function according to oral condition. In a future study, we plan to collect various dental health indicators (Oral Health Impact Profile-14, Xerostomia Inventory, Salivary Flow test) to determine this relationship. Finally, the relationship between respiratory parameters and brain activity should be further evaluated to understand pathophysiological changes due to cognitive impairment caused by oral breathing in a patient study. Quantitative data regarding respiratory parameters such as respiratory rate or abdominal volume should be collected for further analysis.

## 5. Conclusions

In conclusion, we investigated the effect of oral breathing on functional brain activity. It was confirmed that the functional connection decreased significantly during a working memory task in oral breathing rather than nasal breathing. Furthermore, the functional connections of the left cerebellum, and left and right inferior parietal gyrus appeared only during nasal breathing, but not during oral breathing. According to these results, oral breathing can interfere with the efficient performance of working memory. Therefore, brain areas closely related to working memory function were less active during oral breathing, suggesting that prolonged oral breathing could significantly induce impaired cognitive function together with various well-known side effects on the body. These findings also suggest that any solutions for oral breathing should be considered not only for dental care but also for working memory activity.

## Figures and Tables

**Table 1:** The 2-back task response time and accuracy in oral and nasal breathing conditions across all subjects.

Parameter	Breathing	Median (IQR)	Z	*p*
Response time (s)	Oral	0.595 (0.525–0.741)	−0.373 ^a^	0.709
	Nasal	0.627 (0.527–0.726)		
Accuracy (%)	Oral	97.44 (94.87–100)	−1.927 ^b^	0.054
	Nasal	100 (97.44–100)		

*p* values were estimated at a significance level of 5% using the nonparametric Wilcoxon signed-rank test. IQR, Interquartile Range. ^a^ Based on positive ranks. ^b^ Based on negative ranks.

**Table 2:** Each breathing condition (“Nasal” and “Oral”) in 2B > Rest contrast using cluster-level group analysis.

Condition	L/R/M	Region	Cluster (K_E_)	Peak	Peak	Peak MNI Coordinate		
				T	Z score	X	Y	Z
Nasal	R	Inferior parietal gyrus	801	15.18	7.15	36	−44	44
		Caudate nucleus	133	11.33	7.15	16	2	18
		Insula	291	11.26	6.34	30	22	0
		Cerebellum	229	10.44	6.13	28	−58	−28
		Precentral gyrus	184	10.17	6.05	38	0	34
		Middle frontal gyrus	265	8.66	5.59	32	6	58
	M	Vermis	413	9.89	5.97	0	−66	−34
	L	Insula	338	14.49	7.03	−30	20	4
		Precentral gyrus	888	12.68	6.67	−50	8	32
		Inferior parietal gyrus	1295	12.68	6.67	−30	−48	44
		Inferior occipital gyrus	177	11.42	6.38	−40	−62	−8
		Supplementary motor area	865	11.33	6.36	0	10	54
		Cerebellum	127	10.39	6.11	−30	−54	−30
		Middle frontal gyrus	59	8.53	5.55	−44	30	32
		Putamen	153	8.24	5.45	−20	4	12
Oral	R	Inferior parietal gyrus	568	11.48	6.39	36	−46	46
		Putamen	191	10.85	6.23	26	26	2
		Cerebellum	102	8.81	5.64	26	−62	−28
		Superior frontal gyrus	181	8.37	5.49	26	4	58
	L	Inferior parietal gyrus	1201	13.89	6.92	−32	−48	46
		Insula	191	10.95	6.26	−30	22	2
		Middle frontal gyrus	73	10.61	6.17	−30	44	2
		Supplementary motor area	284	9.34	5.81	0	12	52
		Precentral gyrus	655	9.15	5.75	−46	4	30
		Inferior frontal gyrus, triangular part	59	8.72	5.61	−36	26	26

MNI, Montreal Neurological Institute; R, right; L, left; M, medial; 2B, 2-back.

**Table 3:** Nasal breathing vs. oral breathing condition in 2B > Rest contrast using functional connectivity seed to voxel analysis.

Condition	Seed	Region	Cluster (K_E_)	Peak	Peak	Peak MNI Coordinate		
				T	Z Score	X	Y	Z
Nasal > Oral	Cerebellum 6 (L)	Cerebellum 8 (R)	240	5.538	4.771	36	−58	−44
	Inferior parietal gyrus (R)	Parietal operculum cortex (R)	179	4.944	4.364	52	−34	26
		Postcentral gyrus (L)	114	4.617	4.129	−36	−26	48
		Cerebellum 6 (R)	115	4.241	3.847	24	−54	−24
	Inferior parietal gyrus (L)	Postcentral gyrus (L)	239	4.927	4.352	−34	−28	48
	Middle frontal gyrus (L)	Anterior cingulate gyrus	146	4.571	4.095	−2	14	32
		Lateral occipital cortex, (superior division) (L)	202	4.312	3.901	−20	−88	38
Oral > Nasal	Inferior frontal gyrus, triangular part (L)	Postcentral gyrus (R)	123	4.620	4.131	50	−32	56
	Middle frontal gyrus (L)	Postcentral gyrus (R)	215	4.449	4.004	50	−30	54

MNI, Montreal Neurological Institute; R, right; L, left; 2B, 2-back.

## Data Availability

The data presented in this study are available upon request from the corresponding author. The data are not publicly available due to privacy reasons.

## Supplementary Materials

The following are available online at https://www.mdpi.com/article/10.3390/healthcare9060645/s1, Figure S1: A study design, Table S1: General information about motion values of oral and nose breathings, Table S2: Statistical result for mean of motion translation of mouth and nose breathings, Table S3: General characteristics.
