# Supplementary material for: Investigation on the Effect of Oral Breathing on Cognitive Activity Using Functional Brain Imaging
Source: Healthcare (Basel). 2021 May 29;9(6):645. doi: 10.3390/healthcare9060645 (PMC8228257; doi:10.3390/healthcare9060645)
Supplement: Supplementary file 1 [file healthcare-09-00645-s001.zip › healthcare-1166373-SI.pdf]

Supplementary Information for "Investigation for the effect of oral breathing on cognitive activity using functional brain imaging"

Ju-Yeon Jung [1] and Chang-Ki Kang [1,2,3,*]

1 Department of Health Science, Gachon University Graduate School, Incheon 21936, Korea; 95me@naver.com
2 Neuroscience Research Institute, Gachon University, Incheon 21565, Korea
3 Department of Radiological Science, College of Health Science, Gachon University, Incheon 21936, Korea
* Correspondence: ckkang@gachon.ac.kr (C.-K.K.); Tel.: +82-32-820-4110

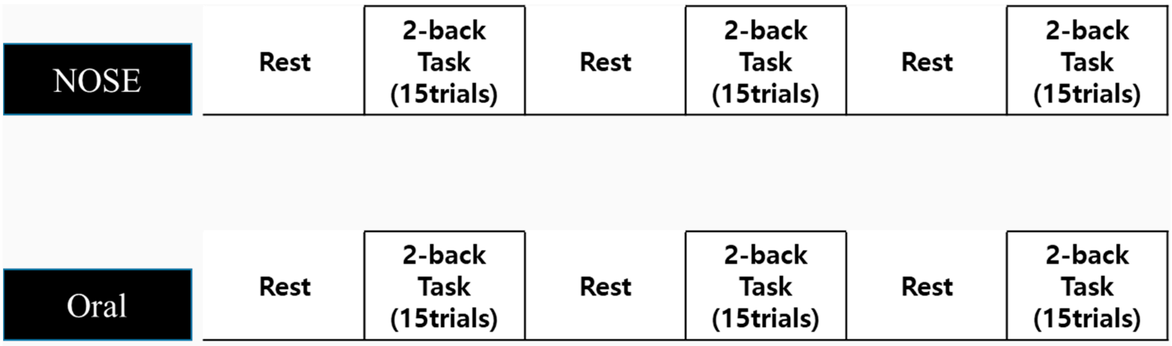

Figure S1. A study design.

Table S1. General information about motion values of oral and nose breathings.

		X	Y	Z	Pitch	Roll	Yaw
Mouth	Mean	0.0187	0.0907	0.0353	0.0013	0.0002	0.0004
	SD	0.1959	0.2656	0.3073	0.0090	0.0039	0.0049
	Max	0.8160	1.2416	1.5249	0.0331	0.0106	0.0246
	Min	-0.9675	-1.6865	-1.6601	-0.0433	-0.0119	-0.0180
Nose	Mean	0.0255	0.0886	-0.0417	0.0003	-0.0009	0.0004
	SD	0.1724	0.1640	0.4055	0.0068	0.0039	0.0041
	Max	0.6214	1.7725	1.6641	0.0302	0.0099	0.0150
	Min	-0.4812	-0.4016	-2.0736	-0.0267	-0.0119	-0.0140

Table S2. Statistical result for mean of motion translation of mouth and nose breathings.

**Motions**	**Axis**	**Statistics (*T*)**	***P* value**
Translation	X	-0.1379	0.8917
	Y	0.0500	0.9666
	Z	0.9489	0.3535
Rotation	Pitch	0.5328	0.5997
	Roll	1.2015	0.2429
	Yaw	0.02757	0.9783

Table S3. General characteristics.

Parameters		Value (n = 22)
Gender (n)†	Male	10
	Female	12
Age (year)*		22.27 ± 1.42
Height (cm)*		170.14 ± 9.28
Weight (kg)*		64.85 ± 9.34

† Valuses are expressed as Number (n).

* Valuses are expressed as Mean ± SD
